# The Role of Markers of Myocardial Damage in Predicting Postoperative Multiple Organ Dysfunction Syndrome and 30-Day Mortality in Patients Undergoing Heart Valve Surgery

**DOI:** 10.3390/jcm15145337

**Published:** 2026-07-08

**Authors:** Piotr Duchnowski, Witold Śmigielski, Krzysztof Kuśmierski

**Affiliations:** Cardinal Wyszynski National Institute of Cardiology, 04-628 Warsaw, Poland; wsmigielski@ikard.pl (W.Ś.); kkusmierski@ikard.pl (K.K.)

**Keywords:** Troponin T, red cell distribution width (RDW), hemoglobin, multiple organ dysfunction syndrome (MODS), valve surgery, risk stratification, EuroSCORE II

## Abstract

**Background:** Multiple organ dysfunction syndrome (MODS) is a serious complication and a leading cause of death in patients undergoing heart valve surgery. The main aim of the present study was to assess the predictive capacity of selected perioperative parameters, including Troponin T levels, to predict the occurrence of postoperative MODS and 30-day mortality in patients undergoing heart valve surgery. **Methods:** This prospective study included a group of patients with hemodynamically severe symptomatic valvular heart disease who underwent valve surgery. The primary endpoint was postoperative multiple organ dysfunction syndrome (MODS), defined as the dysfunction of at least two organs/systems, including cardiogenic shock, perioperative stroke, respiratory failure requiring prolonged mechanical ventilation, and/or postoperative acute kidney injury requiring renal replacement therapy. The secondary endpoint was death during the 30-day follow-up. Logistic regression was used to assess the relationships between the variables. **Results:** In total, 739 patients undergoing valvular heart surgery were included in this study. The primary end point was observed in 45 patients. Preoperative hemoglobin level (*p* = 0.01), red cell distribution width (RDW) (*p* = 0.001) and troponin T level measured on the first day after surgery (TnT II) (*p* < 0.001) were independent predictors of the primary endpoint. EuroSCORE II (*p* = 0.002) and TnT II (*p* < 0.001) were independent predictors of 30-day mortality. **Conclusions:** MODS is a clinical condition that is associated with a high risk of death. Troponin T levels measured within the first 24 h postoperatively may be useful in predicting postoperative MODS and 30-day mortality in patients undergoing heart valve surgery as a complement to commonly used risk calculators.

## 1. Introduction

Cardiac surgery is often the only way to improve functioning and prolong life for patients with valvular heart disease. Unfortunately, this treatment carries a risk of serious postoperative complications [1]. One of the most serious complications in patients undergoing heart valve surgery, and the single leading cause of in-hospital deaths, is multiple organ dysfunction syndrome (MODS). MODS is a progressive, but potentially reversible, dysfunction of at least two systems/organs in the course of an acute and life-threatening disturbance of systemic homeostasis. Given the lack of effective treatment for MODS, early diagnosis and initiation of invasive treatment in the form of mechanical organ support remain the most effective strategies for preventing disease progression and improving treatment outcomes [2,3]. Current cardiac surgery risk assessment systems used to predict outcomes, such as EuroSCORE II and the Society of Thoracic Surgeons score, focus primarily on baseline preoperative variables that assign patients to different risk categories. Perioperative markers are often not considered in prognostic assessments and quality management of care for patients with valvular heart disease in the early postoperative period [4,5]. In the available literature, information on perioperative predictors of serious postoperative events is limited. However, the available literature contains information indicating that parameters of heart muscle damage such as Troponin T determined in the early postoperative period in patients undergoing cardiac surgery may be extremely useful in risk assessment in this group of patients. Troponin T is a protein part of the contractile apparatus of striated muscle. The function of troponin T in all types of striated muscle is the same, but cardiac troponin T (cTnT) differs from the TnT found in skeletal muscle. Therefore, cTnT detected using available high-sensitivity tests in plasma is a highly specific and very sensitive marker of myocardial damage [6].

The main aim of the present study was to assess the predictive capacity of selected perioperative parameters, including Troponin T levels, measured in the immediate postoperative period and on the first postoperative day, in terms of their ability to predict the occurrence of postoperative MODS and 30-day mortality in patients undergoing heart valve surgery.

## 2. Methods

A prospective study was conducted at the Cardinal Wyszyński National Institute of Cardiology in Warsaw in 2014–2021 in a group of patients with severe, hemodynamically symptomatic valvular heart disease who underwent valve replacement/repair surgery. Exclusion criteria were age < 18 years, lack of consent to participate in the study, porcelain aorta, significant atherosclerotic lesions in the carotid arteries detected on ultrasound, autoimmune diseases, chronic inflammatory bowel disease, active infective endocarditis, and active cancer. Plasma troponin T levels collected before the procedure (TnT), immediately after the patient’s arrival at the postoperative ward (TnT I), and on the first postoperative day (TnT II) were determined using the hs-STAT troponin T test (Roche). The procedure was performed under general anesthesia using mechanical ventilation and extracorporeal circulation. The primary endpoint of in-hospital follow-up was postoperative multiple organ dysfunction syndrome, defined as dysfunction of at least two organs/systems, including perioperative brain injury/stroke confirmed by neurological examination and CNS imaging, cardiogenic shock with or without mechanical circulatory support (MCS), respiratory failure requiring prolonged or reintroduced mechanical ventilation, and/or postoperative acute kidney injury requiring renal replacement therapy. The secondary endpoint was death during the 30-day follow-up. Patients included in the study were followed up until discharge from the hospital or until the day of death during their current hospitalization. Each patient gave written consent to participate in the study. The Bioethics Committee of the National Institute of Cardiology in Warsaw approved the study protocol (study number: 2.32/VI/18).

### Statistical Analysis

STATISTICA 12 software (StatSoft Polska Sp. z o.o., Kraków, Poland) was used to perform all the statistical calculations. The statistical significance was set at the level of *p* = 0.05. The median (Q1–Q3) and frequency (%) were used to represent patient characteristics. Comparisons between the assessed groups were performed using the Mann–Whitney U test for quantitative variables and the chi-square test for qualitative variables. Predictors of primary and secondary endpoints were identified using univariate logistic regression analysis. Variables identified as statistically significant in the univariate analysis were included in multivariate logistic regression analysis. Receiver operating characteristic (ROC) curve analysis was used to assess the predictive ability of preoperative and postoperative Troponin T values in predicting the primary and secondary endpoints. Pearson’s linear correlation coefficient was used to calculate the correlation between variables.

## 3. Results

In total, 739 patients undergoing valvular heart surgery were included in this study. Table 1 shows the characteristics of the study groups. Forty-five patients developed multiple organ dysfunction syndrome (MODS), including 37 who required renal replacement therapy, 25 who developed cardiogenic shock (including 18 who received MCS), 16 who had perioperative stroke/CNS damage confirmed by imaging, and 28 who required mechanical ventilation due to respiratory failure. All 45 patients diagnosed with MODS required prolonged inotropic support owing to hemodynamic instability (over 48 h). Table 2 presents the factors that predicted the occurrence of the primary endpoint. In multivariate analysis, preoperative hemoglobin level (OR, 0.779; 95% CI, 0.639–0.949; *p* = 0.01), preoperative red cell distribution width (RDW) (OR, 1.207; 95% CI, 1.093–1.475; *p* = 0.001), and troponin T level measured on postoperative day 1 (hs-TnT II) (OR, 2.557; 95% CI, 1.813–3.605; *p* < 0.001) remained predictors of the primary endpoint. The median TnT II in the group of patients with MODS was 1666 (999–2670) ng/L, which was significantly higher than that in the group of patients without MODS, 600 (343–1111) ng/L (*p* < 0.001). Receiver operating characteristic analysis established a TnT cut-off above 19.8 ng/L, troponin T measured immediately after surgery (TnT I) above 712 ng/L and TnT II above 961 ng/L to predict MODS. Of the 45 patients who developed postoperative MODS, 20 died during the 30-day follow-up period. Co-occurrence of coronary heart disease did not increase the incidence of postoperative MODS. In the study group, 25 patients died within the 30-day follow-up period. In multivariate logistic regression analysis, the independent predictors of 30-day mortality were EuroSCORE II (OR, 1.137; 95% CI, 1.048–1.235; *p* = 0.002) and TnT II (OR, 2.171; 95% CI, 1.436–3.280; *p* < 0.001). In turn, receiver operating characteristic analysis established a cut-off value of TnT above 19.8 ng/L, TnT I above 729 ng/L, and TnT II above 1275 ng/L for predicting 30-day mortality. The area under the receiver operator characteristic curve for 30-day mortality for EuroSCORE II is 0.699 (95% CI 0.664–0.732) and for TnT II is 0.766 (0.733–0.797). A significant correlation (*p* < 0.05) was found between the preoperative left ventricular ejection fraction (LV EF) and TnT (r = −0.16), NT-proBNP (r = −0.31) and RDW (r = −0.2) levels, as well as between TnT and TnT I (r = 0.21), and between the duration of extracorporeal circulation and the level of TnT I (r = 0.34) and TnT II (r = 0.06).

## 4. Discussion

Multiple organ dysfunction syndrome is one of the most serious complications that can occur in the postoperative period in patients undergoing cardiac surgery for valvular heart disease. It is a cause of prolonged hospitalization, increased treatment costs, and a major contributor to poor prognosis. Tissue damage caused by hypoxia is one of the primary causes of multiple organ dysfunction syndrome. Tissue hypoxia is usually a consequence of one of three underlying causes that may coexist: reduced cardiac output, reduced hemoglobin levels, and/or impaired oxygen uptake by target cells [7,8,9]. In this study, multiple organ dysfunction syndrome (MODS) occurred in 45 patients, 20 of whom died during the 30-day follow-up period. It is noteworthy that all 45 patients in MODS required prolonged (over 48 h) support with positive inotropic drugs. Postoperative hemodynamic instability is a fairly common complication in patients undergoing open-heart surgery. Prolonged hemodynamic instability can lead to multiple organ dysfunction, resulting in prolonged intensive care unit (ICU) stay and significant postoperative morbidity and mortality [7,9]. In patients with features of developing MODS, early use of mechanical support of organs such as hemodiafiltration, mechanical circulatory support, or mechanical ventilation allows for the correction of volumetric, respiratory, and metabolic disorders and improves perfusion and oxygenation of peripheral tissues, thus improving conditions for tissue regeneration, which may contribute to improving the prognosis in this extremely high-risk group of patients [10]. Utilizing knowledge about the predictors of postoperative MODS is crucial because it allows for the identification of patients at particular risk. This is helpful in the qualification period of patients for the procedure, optimizing the choice of procedure and, if possible, postponing the procedure date in order to compensate for disturbances, including blood morphology. In turn, in patients undergoing surgery, special supervision of patients, early correction of disorders and implementation of possible treatment. The results of this study indicate that preoperative morphotic parameters such as hemoglobin and RDW, i.e., markers defining oxygen transport capabilities and physiological reserves, were independent predictors of postoperative MODS. Furthermore, troponin T measured in the early postoperative period was also an independent predictor of MODS. The median TnT II level in the MODS group was significantly higher than that of the group of patients without MODS. It is worth noting that the level of Troponin T determined on the first day after surgery was the only parameter, apart from the risk calculation result of the EuroSCORE II calculator, which was the only independent predictor of 30-day postoperative mortality.

To date, the use of serum troponin T levels assessed in the early postoperative period as a prognostic variable in patients undergoing cardiac surgery for valvular heart disease has not been widely investigated. Current risk-scoring systems used to predict outcomes after cardiac surgery, such as EuroSCORE II or STS, focus primarily on preoperative “core variables” that profile patients into different risk categories [4,5]. The results of the above study, as well as the authors’ previous studies, indicate that the determination and use of troponin T in the early period may be very useful in predicting serious complications, including postoperative hemodynamic instability, MODS and early postoperative death in patients undergoing cardiac surgery due to valvular heart disease [6,7,9,11,12,13,14]. Significant increases in troponin T levels in the postoperative period in patients undergoing cardiac surgery may have various causes. The demonstrated correlation between preoperative LV EF and TnT and NT-proBNP, as well as between TnT and TnT I, and also between time of extracorporeal circulation and TnT I and TnT II, may indicate that the overloaded/damaged heart muscle due to valvular heart disease is particularly sensitive to the unphysiological conditions encountered during cardiac surgery (especially the use of cardioplegia, extracorporeal circulation and perioperative blood loss), which may result further in worsening myocardial damage and lead to postoperative hemodynamic instability, and consequently, hypoxia and peripheral tissue damage, potentially leading to organ dysfunction. It appears, therefore, that troponin T measured in the early postoperative period, regardless of commonly used risk calculators such as EuroSCORE, may be a biomarker whose use will contribute to improved risk stratification in patients in the early postoperative period. Furthermore, a broader understanding of preoperative parameters such as LVEF, TnT, NT-proBNP, as well as blood count parameters such as hemoglobin level and RDW, will help optimize the choice of procedure type and timing, which may translate into improved prognosis in some patients [15,16,17,18,19,20].

This was a single-center study with a limited number of patients and a short follow-up period. The lack of external validation may limit the generalizability and reliability of this model across different patient populations and clinical settings. Therefore, future studies with larger patient numbers, expanded centers, and extended follow-up may confirm our findings. It is also necessary to clarify all the reasons for the increase in troponin T levels in the early postoperative period because their knowledge and reduction may also influence the positive effect of treatment.

## 5. Conclusions

Multiple organ dysfunction syndrome (MODS) is one of the most serious complications that can occur in the early postoperative period and carries a high risk of death. In this study, among patients with postoperative MODS, 30-day mortality occurred in 20 patients. The study results indicate that preoperative blood count parameters such as hemoglobin and RDW, as well as troponin T levels measured within the first 24 h, may be useful in predicting postoperative and 30-day mortality, independent of commonly used risk calculators.

## Figures and Tables

**Table 1:** Baseline characteristics of the study population (*n* = 739).

Characteristics of Patients (*n* = 739)	Values All Patients	Patients with MODS (*n* = 45)	Patients Without MODS (*n* = 694)	*p*-Value
Age, years	65 (58–71)	73 (63–77)	65 (58–71)	<0.001
Male: men, *n* (%)	410 (55)	20 (44)	390 (56)	0.07
BMI, kg/m^2^	27 (25–30)	27 (25–30)	25 (25–30)	0.34
EuroSCORE II, %	2.2 (1.3–3.6)	3.6 (2.4–6.7)	2.1 (1.2–3.5)	<0.001
LV ejection fraction, %	60 (53–65)	58 (50–65)	60 (53–65)	0.36
Coronary artery disease, *n* (%)	186 (25)	15 (33)	171 (24)	0.19
Atrial fibrillation, *n* (%)	282 (39)	29 (64)	253 (36)	<0.001
Diabetes mellitus, *n* (%)	127 (15)	12 (26)	115 (16)	0.08
Hemoglobin, g/dL	13.7 (12.7–14.6)	12.8 (11.3–13.6)	13.7 (12.8–14.7)	<0.001
GFR, mmol/L	64 (57–79)	58 (41–72)	64 (58–80)	<0.001
RDW,%	13.8 (13.2–14.6)	15.1 (14.4–17)	13.7 (13.2–14.4)	<0.001
TnT, ng/L	12.4 (7.5–21)	22.7 (20–38)	12 (7.5–20)	<0.001
NT-proBNP, pg/mL	898 (330–1966)	2948 (671–4524)	826 (303–1880)	<0.001
CRP, mg/dL	0.21 (0.1–0.43)	0.46 (0.25–0.59)	0.2 (0.1–0.4)	<0.001
Postoperative characteristics of patients				
Rethoracotomy, *n* (%)	82 (11)	19 (42)	63 (9)	<0.001
Stroke, *n* (%)	23 (3.1)	16 (35)	7 (1)	<0.001
Renal replacement therapy, *n* (%)	50 (6.7)	37 (82)	13 (2)	<0.001
Shock, *n* (%)	34 (4.6)	25 (55)	9 (1)	<0.001
Hospital stay after surgery, day	10 (8–16)	30 (13–47)	10 (8–15)	<0.001
30-day mortality, *n* (%)	25 (3.3)	20 (44)	5 (1)	<0.001
TnT I, ng/L	584 (341–997)	985 (605–1781)	568 (336–956)	<0.001
TnT II, ng/L	643 (355–1237)	1666 (999–2670)	600 (343–1111)	<0.001
Cross-clamp time, min	80 (60–116)	116 (78–155)	71 (60–113)	0.1
Bypass time, min	107 (78–154)	129 (115–215)	98 (77–135)	0.02
Type of operation				
Single valve surgery, *n* (%)	405 (54)	7 (15)	398 (57)	
Multiple valve surgery, *n* (%)	243 (32)	29 (64)	214 (29)	
Valve surgery + CABG, *n* (%)	91 (12)	9 (20)	82 (11)	

Data are expressed as median (Q1–Q3) and frequency (%). Abbreviations: CABG, coronary artery bypass graft; CRP, C-reactive protein; GFR, glomerular filtration rate; LV, left ventricle; ICU, intensive care unit; NT-proBNP, N-terminal of the prohormone brain natriuretic peptide; RDW, red cell distribution width; TnT, preoperative troponin T; TnT I, troponin T measured on 0 day after surgery; TnT II, troponin T measured on the first day after surgery.

**Table 2:** Analysis of predictive factors for the occurrence of the primary endpoint.

	Univariate Analysis			Multivariate Analysis		
Variable	Odds Ratio	95% Cl	*p*-Value	Odds Ratio	95% Cl	*p*-Value
NT-proBNP, pg/mL	1.845	1.403–2.425	<0.001			
RDW, (%)	1.432	1.257–1.633	<0.001	1.207	1.093–1.475	0.001
Hemoglobin, g/dL	0.675	0.565–0.806	<0.001	0.779	0.639–0.949	0.01
GFR, mL/min/1.73 m^2^), *n* (%)	0.958	0.940–0.976	<0.001			
CRP, mg/dL	1.854	1.344–2.559	<0.001			
TnT, ng/L	2.009	1.427–2.827	<0.001			
TnT I, ng/L	2.313	1.628–3.287	<0.001			
TnT II, ng/L	2.670	1.948–3.658	<0.001	2.557	1.813–3.605	<0.001

Abbreviations: CRP = C-reactive protein, GFR = glomerular filtration rate, NT-proBNP = n-terminal of the prohormone brain natriuretic peptide, RDW = red cell distribution width, TnT = preoperative troponin T, TnT I = troponin T measured immediately after surgery, TnT II = troponin T measured one day after surgery.

## Data Availability

The original contributions presented in this study are included in the article. Further inquiries can be directed to the corresponding author.
